# The role of teledermatology in Mohs micrographic surgery: a review

**DOI:** 10.1007/s00403-024-02851-2

**Published:** 2024-04-16

**Authors:** Yanci A. Algarin, Dana Jaalouk, Anika Pulumati, Keyvan Nouri

**Affiliations:** 1 Department of Dermatology, Eastern Virginia Medical School, Norfolk, VA USA; 2 Department of Dermatology and Cutaneous Surgery, University of Miami Leonard M. Miller School of Medicine, Miami, FL USA; 3 Department of Dermatology, University of Missouri-Kansas City School of Medicine, Kansas City, MO USA

**Keywords:** Teledermatology, Mohs micrographic surgery, Telemedicine, Dermatologic surgery, Telepathology

## Abstract

This paper explores the role of teledermatology (TD) in Mohs micrographic surgery (MMS) at various stages of patient care. The study aims to assess the benefits, limitations, and patient experiences surrounding TD integration into MMS practices. We conducted a PubMed search using keywords related to TD and MMS, categorizing selected articles into pre-operative, intra-operative, and post-operative stages of MMS. TD reduced waiting times (26.10 days for TD compared to 60.57 days for face-to-face [FTF]) and consultation failure rates (6% for TD vs. 17% for FTF) for MMS preoperative consultations. It also shortened time to treatment by two weeks and led to notable travel savings (162.7 min, 144.5 miles, and $60.00 per person). Telepathology facilitated communication and decision-making during MMS, improving accuracy and efficiency, especially in challenging cases requiring collaboration where physical presence of another surgeon or pathologist is not feasible. Telepathology definitively diagnosed benign lesions and malignant tumors in 81.8% of cases (18/22). Additionally, there was a 95% agreement between conventional light microscopy diagnosis and telepathology in tumors (19/20), and 100% agreement for all 20 Mohs frozen section consultations. For post-operative follow-up, telephone follow-up (TFU) and text messaging proved effective, cost-efficient alternatives with high patient satisfaction (94% in New Zealand and 96% in the U.K.) and early complication identification. This study underscores TD’s multifaceted benefits in MMS: enhanced patient experience preoperatively, improved communication during surgery, and cost-effective postoperative follow-up. Limitations include the financial expense and technical issues that can arise with TD (connectivity problems, delays in video/audio transmission, etc.). Further studies are needed to explore emerging TD modalities in post-operative patient management. The integration of TD into MMS signifies a progressive step in dermatological care, offering convenient, cost-effective, and better solutions with the potential to enhance patient experiences and outcomes.

## Introduction

Teledermatology (TD) is the practice of using technology (video conferencing, phone calls, digital cameras, and smartphone apps) for diagnosing and treating dermatological conditions remotely [1]. A survey of 115 Mohs surgeons revealed that 86.1% utilized TD during the pandemic for purposes including post-surgery management (77.4%), spot checks (60.9%), and surgical consultations (59.1%). With a high patient acceptance rate (73.1%) and nearly half of the surgeons (49.5%) planning to continue its use long-term, understanding the benefits, challenges, and patient experience is crucial for effectively integrating TD into Mohs surgery practices [2].

Although Mohs micrographic surgery (MMS) is not categorized as “emergency care”, COVID-19 pandemic-related delays led to increased local tumor spread and upstaging of skin cancers [3]. Such delays increased case complexity for 68.5% of surgeons, and exacerbated emotional distress for 76% of patients [3]. Considering these risks, it is crucial that Mohs surgeons are able to proceed with treatment, while still maintaining patient safety. Our paper explores TD’s multifaceted role in MMS, assessing its pre-, intra-, and post-operative applications. We evaluate its benefits, challenges, and areas needing further research, aiming to shed light on how TD can revolutionize MMS.

## Methods

### Search strategy

We conducted a comprehensive search on PubMed from 1998 to 2023 using the following keywords and search terms: “Mohs micrographic surgery” AND one of the following search terms: “Telemedicine” or “Teledermatology” or “Telepathology” or “Telephone” or “Virtual” or “Telehealth” or “Remote” or “Mobile health.” We screened all identified articles for relevance to our review topic, selecting those meeting our inclusion criteria for further analysis. References from relevant articles were also used to locate more articles for our study.

### Inclusion and exclusion criteria

Our inclusion criteria comprised English articles published in peer-reviewed journals that discussed the application of TD in MMS. However, non-peer-reviewed articles, including conference abstracts or non-scientific literature, not written in English and not specifically addressing the use of TD within MMS were excluded.

## Teledermatology and MMS

### Preoperative consultation

TD has not been studied extensively as a tool for preoperative consultation for MMS, but shows significant promise in enhancing the patient experience. Fernandez et al.‘s multicenter pilot study revealed that TD significantly reduced preoperative waiting times for non-melanoma skin cancer (NMSC) from 60.57 days (face-to-face visits) to 26.10 days (TD), demonstrating high diagnostic reliability (κ = 0.86) to histopathologic diagnoses and strong agreement in surgical planning (κ = 0.75). This indicates its effectiveness as a preoperative tool [4]. Moreover, Lee et al. found a decrease in consult failure rates (CFRs) from 17 to 6% with TD compared to face-to-face (FTF) consultations. The CFR for each modality was calculated by dividing the number of lesions resulting in failed consults by the total lesions consulted, representing the percentage of lesions that led to failed consults. TD also decreased the time to treatment by two weeks, increased the percentage of lesions treated within 60 days, and resulted in average travel savings of 162.7 min, 144.5 miles, and $60.00 per person [5]. Furthermore, Nicholson et al. found that outcomes of pre-operative consultations conducted through TD were comparable to FTF consultations, with no significant difference in the mean number of MMS stages or sections between the two groups. Consistently, the reconstructive strategy established during the TD consultation was executed as planned, eliminating the need for external surgical referrals, proving an effective alternative to FTF consultations [6]. These findings demonstrate TD as an effective and accurate preoperative tool for MMS, enabling patients to avoid unnecessary hospital visits and experience reduced waiting times [5, 6].

Du et al. analyzed 1,449 participants’ preferences for TD versus FTF consultations for MMS, which included 1,394 Amazon Mechanical Turk (MTurk) responses and 55 in-person surveys [7]. While 82.1% of MTurk respondents preferred FTF, this preference decreased to 58.3% with a $100 cost (*p* < 0.01) and further to 43.5% with both a $100 cost and an hour wait time for FTF, compared to a $50 cost for TD (*p* < 0.01). Despite a preference for FTF assessments and a shared belief that FTF visits would improve a surgeon’s skills among both groups, cost and wait time appear to weigh more heavily in decision-making over quality of care. TD is more preferred by Medicaid or Medicare beneficiaries, previous skin cancer patients, and lower-income groups, indicating its appeal to those facing socioeconomic barriers to healthcare access. Ultimately, patients prefer FTF appointments for skin cancer reconstruction care, but this preference shifts towards TD when considering increased costs and longer waiting times [7]. However, the study’s method of assessing travel burden, which used patient-selected town type and rurality as proxies, may not accurately reflect actual travel distances and costs. Additionally, while the survey results are consistent with those from the online cohort, the external validity of conclusions drawn from multivariable analyses remains uncertain [7].

TD has many apparent advantages for patients and Mohs surgeons, but also has some limitations. Technical challenges such as delays in page loading, report transmission issues, and risks of losing work due to glitches necessitate robust information technology support.

The cost of investing and maintaining TD equipment and technology support is another significant consideration. In video consultations, suboptimal image quality can impede effective lesion evaluation [2]. Additionally, elderly patients may face challenges in using TD, including difficulties in adequately displaying lesions or adhering to scheduled times [8–10]. While physical palpation is impossible using TD services, its absence has not been significantly noted as a limitation by dermatologists [11]. Further large-scale studies and randomized trials are needed to comprehensively assess the clinical and economic impacts of TD compared to traditional methods.

Evidence suggests that TD is a promising tool for enhancing the patient experience, and is an effective and generally accurate preoperative tool for Mohs surgeons. Some studies have demonstrated its effectiveness in reducing wait times, CFR, and travel burdens for patients.^6,7^ However, the benefits of TD are largely related to logistical aspects rather than direct improvements in surgical or clinical outcomes [7]. Notably, patients facing socioeconomic barriers to healthcare access showed a strong preference for TD highlighting its potential to bridge healthcare disparities. Ultimately, TD serves well as an adjunctive tool for preoperative consultations in Mohs surgery, enhancing patient accessibility and convenience. Mohs surgeons should integrate TD alongside traditional care methods, leveraging its strengths in facilitating access and improving patient experience, rather than as a comprehensive alternative for clinical or surgical enhancements.

### Intraoperative guidance

Telepathology involves transmitting histopathologic images to a remote pathologist using imaging systems [12]. Telepathology is a versatile tool with many applications such as remote primary pathology diagnosis, consultations in complex cases, and remote teaching [12]. However, its use for intraoperative consultation during MMS has not been extensively studied but is gaining significant interest [13].

In their study, Sukal et al. utilized a dynamic telepathology system, connecting an automated microscope to a PC with Windows NT and MedMicro software [13]. This setup linked the Mohs laboratory with a remote viewing site, and efficiently scanned histopathologic slides in 1.5 min, creating virtual images for analysis. In 25 cases with uncertainties in tumor histology, telepathology confirmed tumor histology and provided prognostic insights. In 18 of 22 cases (81.8%), it accurately distinguished between benign lesions and malignant tumors, guiding subsequent MMS procedures. However, in four cases, telepathology faced challenges in definitively differentiating benign from malignant lesions, and an additional Mohs stage was performed [13]. In a separate study using telepathology during MMS for lentigo maligna (*n* = 96), clearance rates aligned with previous MMS cases for this condition (97%) [14]. The significance of advanced digital camera technology has also been highlighted, particularly for its role in expediting image transmission. This allowed a remote pathologist to swiftly identify a benign lesion within 15 min of receiving a digitally transmitted image, effectively preventing surgical delays [15].

Rapid and accurate decision-making during intraoperative evaluation of frozen sections is critical in surgical procedures. A study at Memorial Sloan-Kettering Cancer Center demonstrated the efficacy of a dynamic telepathology system in this context. The system was used to assess 50 fixed-tissue slides of NMSC for diagnosis, 40 frozen-section slides for tumor presence, and 20 for intraoperative consultations. The study found complete concordance between telepathology and conventional light microscopy for all 110 slides, underscoring the accuracy of telepathology [12]. Further validation came from a study utilizing iChat AV video conferencing for real-time transmission of histologic images and audio from a MMS lab to a remote dermatopathologist [16]. This setup, compatible with Apple Macintosh platforms, allowed for immediate consultation. There was a 95% (19/20) agreement in tumor diagnosis between dynamic telepathology and conventional microscopy, and a 100% agreement for all 20 Mohs frozen section consultations. The ability of this system to capture and transmit both video and audio was crucial in ensuring high-quality, accurate histologic evaluations and effective communication between the surgeon and the dermatopathologist [16]. Additionally, telepathology has practical applications in urban settings where it overcomes logistical challenges like specimen transport delays, as highlighted by Nehal and colleagues [12].

Another emerging application in TD is smart glasses. A recent study at Loma Linda University explored the use of smart glasses in MMS [17]. These hands-free, voice-activated devices, which integrate into standard eyeglasses and display information on a translucent screen, proved beneficial in surgical settings. The study demonstrated their utility in capturing and immediately sharing photographs and videos of post-excision defects with surgeons. Notably, the smart glasses also facilitated real-time video conferencing for teleconsultations, enabling surgeons to remotely participate in case discussions and decision-making processes, all while maintaining the sterility of the surgical field [17].

The studies reviewed highlight the effectiveness of various TD applications in streamlining MMS, enhancing efficiency and convenience. However, it’s important to recognize certain limitations in their intraoperative use. In the U.S., where the Mohs surgeon often also functions as the dermatopathologist, the necessity of telepathology for intraoperative communication is less pronounced. Additionally, the cost of telepathology equipment presents a barrier, particularly in resource-limited settings. Addressing these challenges, innovative, cost-effective solutions have emerged. For instance, Kantor’s technique, involving a plastic binocular adapter to attach a smartphone to a microscope, enables slide visualization on the phone and wireless projection, circumventing the need for expensive equipment and internet connectivity [18, 19]. This approach underscores the potential to mitigate technological and financial constraints. Despite these advances, the limited clinical use of these TD applications suggests a need for further research to fully understand their benefits and limitations before broader implementation.

### Postoperative follow-up

FTF postoperative checks are the standard of post-surgical care. However, this standard practice places strain on clinical schedules and financial resources, potentially leading to higher expenses for healthcare providers and patients [20]. Telephone follow-up (TFU) provides a practical alternative, offering a cost-effective and efficient method in recognizing post-surgical complications, improving patient satisfaction, and streamlining clinic operations [21–26]. TFU has been shown to improve patient adherence to treatments, reduce unnecessary emergency department visits, and allow for early addressing of postoperative issues, potentially leading to better patient outcomes [22, 27, 28].

Hafiji et al. revealed high patient satisfaction with TFU post-MMS (94% in New Zealand and 96% in the U.K.) [22]. The study involved surgeons calling patients on the evening of their surgery. Additionally, fewer patients in the TFU group needed post-operative contact with their surgeon, compared to 7% in the control group who needed follow-up for issues addressable by TFU [22]. Jeyamohan et al. found that all patients (*n* = 43) reported improvement of their symptoms following TFU. Patients demonstrated a future preference for using photographs as opposed to a FTF visit, citing convenience and rapid access to evaluation and care. Other benefits included a high level of patient satisfaction, limiting unnecessary in-office visits, and empowerment of patients in their healthcare delivery [10].

Vance et al. and Bednarek et al. conducted studies evaluating the impact of TFU in post-MMS care [29, 30] Vance et al. examined patient and scar satisfaction in a randomized, single-blind study (*n* = 104), comparing a group receiving TFUs with a non-TFU group. Results showed no significant difference in satisfaction at suture removal and three-month follow-ups (*p* = 0.80 and *p* = 0.51, respectively), although patients who received calls preferred TFU over other communication methods [29]. Bednarek et al. focused on the optimal timing for TFU in a 400-patient study, assessing patient satisfaction and early complication detection. The study found that TFUs on the evening of surgery effectively identified active pain, but were not superior in detecting severe pain or bleeding complications. Overall, no significant differences in patient satisfaction were observed across different TFU timings. The authors suggest that TFUs on the evening of surgery may offer better opportunities for guiding patients in pain management [30].

Forbes et al. also revealed that TFU is a feasible and effective strategy for the detection of potential postoperative MMS complications (*n* = 349) [31]. There were reports of major complications in only 0.4% (*n* = 1) for bleeding and 0.4% (*n* = 1) for infection which were treated by the operating surgeon. Minor complications were also noted, but required no further treatment. The authors concluded that TFUs post-MMS enable a clearer understanding of patient complications without compromising patient safety. Furthermore, the calls were concise, averaging approximately three minutes [31].

Text messaging is another effective TD method for patient interaction and education. A randomized trial by Hawkins et al. divided 90 MMS patients into four groups to receive various combinations of educational and postoperative follow-up communications: videos plus text messages, videos only, text messages only, and a control group receiving standard nurse-led instructions with a supplementary written guide [32]. The study observed a 19% decrease in patient anxiety following the video (*p* = 0.00062) and a strong preference among patients to receive future wound care instructions via text messages (*p* = 0.0001). An impressive 91% of participants rated the text message service as “very helpful” or “helpful” [32].

Overall, data surrounding the use of post-operative TD in MMS is limited [10, 22, 29–31]. Most studies evaluated the impact of TFU on patient satisfaction and/or identification of complications. Regarding patient satisfaction, the results from ​​Bednarek et al. and Vance et al. diverge from the findings of Hafiji et al., demonstrating that high patient satisfaction with TFU was not statistically significant compared to controls. Concerning complications, Bednarek et al. and Forbes et al. both revealed that TFU can be used as a tool to aid in early identification of complications [29, 30]. Moreover, an overwhelming majority of patients found postoperative text messaging helpful [31].

Although none of the studies explicitly evaluated potential cost reductions for patients, it is reasonable to expect reduced expenses due to less time commitment and travel [30]. TFU offers immediate reassurance or guidance on postoperative symptoms without compromising patient safety, and can address patient concerns before complications escalate [30]. Early identification of complications also allows for timely intervention during the post-surgical visit. Some suggest that reducing complication rates and minimizing in-office visits could liberate additional resources, thereby enhancing the availability of MMS services [30, 32].

Routine TFU could lead to shorter waiting lists, increase availability for new referrals, and decrease non-attendance [32]. Despite low complications in MMS, TFU remains valuable because it helps alleviate patient anxiety and increases patient satisfaction [33, 34]. Additionally, post-operative text reminders can improve patient adherence to wound care instructions and reduce treatment interruptions [35]. There is a research gap regarding video conferencing and other TD modalities in post-MMS care, suggesting an area of focus for future studies.

## Conclusion

The role of TD in MMS is multifaceted and offers benefits at all stages of patient care.

Preoperatively, TD has reduced wait times, CFR, and travel burdens, enhancing the patient experience. Intraoperatively, telepathology and smart glasses demonstrated improved communication and decision-making, with potential to enhance the accuracy and efficiency of MMS procedures. Postoperatively, follow-up through telephone calls and other digital methods offered a cost-effective and efficient means of monitoring patients, reducing the need for in-person visits.

It is evident that the integration of TD services into MMS is a step towards advancing dermatological care. As the healthcare landscape continues to evolve, implementing TD in clinical practice can lead to more convenient, cost-effective, and better healthcare solutions for patients, ultimately enhancing patient experiences and outcomes.

## Data Availability

No datasets were generated or analysed during the current study.
